# Circular RNAs Could Encode Unique Proteins and Affect Cancer Pathways

**DOI:** 10.3390/biology12040493

**Published:** 2023-03-24

**Authors:** Francesca Crudele, Nicoletta Bianchi, Anna Terrazzan, Pietro Ancona, Antonio Frassoldati, Paolo Gasparini, Adamo P. D’Adamo, Dimitrios Papaioannou, Ramiro Garzon, Anna Wójcicka, Paweł Gaj, Krystian Jażdżewski, Jeffrey Palatini, Stefano Volinia

**Affiliations:** 1 Department of Translational Medicine, University of Ferrara, 44121 Ferrara, Italy; crdfnc@unife.it (F.C.);; 2 Genetics Unit, Institute for Maternal and Child Health, Scientific Institute for Research, Hospitalization and Healthcare (IRCCS) Burlo Garofolo, 34137 Trieste, Italy; 3 Laboratory for Advanced Therapy Technologies (LTTA), University of Ferrara, 44121 Ferrara, Italy; 4 Department of Oncology, Azienda Ospedaliero-Universitaria St. Anna di Ferrara, 44124 Ferrara, Italy; 5 Laura and Isaac Perlmutter Cancer Center, New York University School of Medicine, NYU Langone Health, New York, NY 10016, USA; 6 Division of Hematology and Hematological Malignancies, University of Utah, Salt Lake City, UT 84112, USA; 7 Warsaw Genomics INC, 01-682 Warszawa, Poland; 8 Human Cancer Genetics, Biological and Chemical Research Centre, University of Warsaw, 02-089 Warsaw, Poland; 9 Genomics Core Facility, Centre of New Technologies, University of Warsaw, 02-097 Warsaw, Poland; 10 CNBCh, Biological and Chemical Research Centre, University of Warsaw, 02-089 Warsaw, Poland

**Keywords:** CircRNA, RNA-Seq, translation, tumor, AML

## Abstract

**Simple Summary:**

In this study, we investigated human genes encoding circular RNAs (circRNAs) in order to shed light on their functional role, which is still under debate. We identified 183 genes encoding circRNAs differentially expressed in cancer tissues with a novel coding potential. Our data suggest that circRNAs might directly affect cellular and systemic processes in cancer by generating novel members of the human proteome.

**Abstract:**

circRNAs constitute a novel class of RNA, generally considered as non-coding RNAs; nonetheless, their coding potential has been under scrutiny. In this work, we systematically explored the predicted proteins of more than 160,000 circRNAs detected by exome capture RNA-sequencing and collected in the MiOncoCirc pan-cancer compendium, including normal and cancer samples from different types of tissues. For the functional evaluation, we compared their primary structure and domain composition with those derived from the same linear mRNAs. Among the 4362 circRNAs potentially encoding proteins with a unique primary structure and 1179 encoding proteins with a novel domain composition, 183 were differentially expressed in cancer. In particular, eight were associated with prognosis in acute myeloid leukemia. The functional classification of the dysregulated circRNA-encoded polypeptides showed an enrichment in the heme and cancer signaling, DNA-binding, and phosphorylation processes, and disclosed the roles of some circRNA-based effectors in cancer.

## 1. Introduction

CircRNAs are covalently closed molecules with a tissue- and cell-specific expression, whose biogenesis is specifically regulated [1]. They may exert biological functions by acting as microRNAs (“sponges”) and as protein inhibitors (“decoys”), but they could also be translated into proteins. Recent advances in RNA-sequencing (RNA-seq) and circRNA-specific decoding tools allowed for their quantification and characterization, leading to the identification of tens of thousands of potential circRNAs transcribed from the human genome [2,3] and modulated in breast cancer and other tumors, such as in leukemias [4,5]. In this context, we recently performed the first large-scale study of circRNAs considering over 400 total RNA-seq samples from patients with acute myeloid leukemia (AML) [6]. Nevertheless, the full functional role of circRNAs in cancer is still under debate [7], and several studies have asserted that circRNAs can act as templates for translation. Indeed, Abe et al. demonstrated that a pool of circRNAs comprised boundless Open Reading Frames (ORFs) that could be translated into a protein concatemer by a mechanism called “rolling circle amplification” [8]. Furthermore, Chen et al. confirmed the cytoplasmic localization of circRNAs in eukaryotic cells [9]. Different research groups have explained two cap-independent mechanisms of circRNAs translation: the internal ribosome entry sites (IRES) and the N6-methyladenosines (m6A)-mediated translation [10,11]. Currently, an increasing number of studies have taken into account the coding potential of circRNAs, as well as the role of the peptides encoded by both circRNAs and long non-coding RNAs, as it occurs in glioblastoma [12,13,14], liver cancer [15], and neurodegenerative diseases [16]. Here, we investigated the coding potential of circRNAs sourced by MiOncoCirc, a pan-cancer compendium of more than 160,000 cancerous circRNAs detected through a poly(A)-independent method and gene-body targeting, the exome capture RNA-seq [5,17], focusing on a restricted set of circRNAs with a high potential to generate novel isoforms.

## 2. Materials and Methods

### 2.1. Cancer circRNA Selection

The MiOncoCirc dataset (https://mioncocirc.github.io/download/, accessed on 15 June 2022) includes RNA-Seq data from a large number (*n* = 2036) of cancer samples, derived from several types of tissue (prostate, breast, lung, pancreatic, liver, etc.) [18,19,20] (Table S1). After excluding the read-through circRNAs located within intergenic sequences, we selected circRNAs expressed in at least 40 different samples. As alternative splicing events frequently occur within each circRNA, we considered only those at least 150 nucleotides long, characterized using their genomic coordinates in GENCODE (v.33) to determine all of the different spliced isoforms (*n* = 56,819). Using TransDecoder (v.5.5.0), we predicted the circular ORFs (circORFs) encoding for polypeptides at least 50 amino acids (aa) long, starting with a methionine and ending with a stop codon for each circRNA transcripts.

### 2.2. In Silico Characterization of Polypeptides Predicted from circRNAs

Using protein BLAST version 2.9.0 (https://rcc.uchicago.edu/docs/software/modules/blast/midway2/2.9.0.html, accessed on 15 June 2022) we determined the correspondence between the polypeptides encoded by the longest circORF and the cognate linear isoforms obtained from Ensembl 101 (https://www.ensembl.info/known-bugs/ensembl-101/, accessed on 15 June 2022), using a threshold E-value of 1.0 × 10^−10^. Therefore, we underline that no circRNAs had multiple putative ORFs. To restrict the cognate field, we aligned only proteins from the Consensus Coding Sequence (CCDS) database (https://www.ncbi.nlm.nih.gov/, accessed on 15 June 2022). We developed a Python script to isolate all circRNAs encoding proteins with a mismatch of at least one amino acid at the N- and/or C-terminus compared with their respective CCDS isoforms; these circORF encoded proteins were thus annotated accordingly: “canonical” or “internal” for the starting methionine, and/or with “premature” or “canonical” C-terminus. Lastly, the new peptides at the carboxy- and ammino-terminus were annotated as C-term and N-term, respectively. 

### 2.3. The Domain Structure of circRNA Encoded Proteins

**Figure 1:**
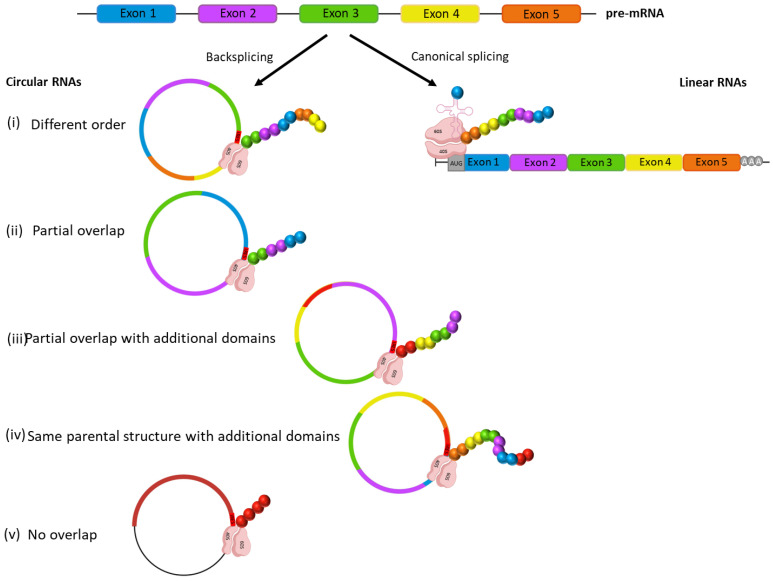
Schematic representation of the different circRNA proteins generated, focusing on their domain composition in comparison with the linear isoform.

The domain composition of the circRNA proteins was globally investigated using HMMER HmmScan (https://www.ebi.ac.uk/Tools/pfa/hmmer_hmmscan/, accessed on 15 June 2022). We compared the domains of each circRNA protein with those of their cognate linear isoforms (GENCODE v. 33 (https://www.gencodegenes.org/human/release_33.html, accessed on 15 June 2022). We developed a Python script to identify the predicted circular proteins with domain mismatches in comparison with the linear isoforms, considering the following: (i) different order of the domains, (ii) partial overlap (missing one or more domains when compared to the parental isoforms), (iii) partial overlap with one or more additional domains, (iv) same parental domain structure with additional domains, or (v) no overlap with the domain structure of all cognate linear isoforms (Figure 1).

### 2.4. Expression Profile of circRNAs with Unique Protein-Coding Potential in Cancer

To investigate the cancer expression of circRNAs predicted with uniquely encoded polypeptides, we used the MiOncoCirc RNA-Seq data collection of clinical samples [5], cell lines, and normal tissues (*n* = 2036) [18,19,20], annotated in Table S2. Data were expressed as log_2_ reads per million (RPM) and normalized by quantile. A large number of samples showed overall very low levels of circRNAs in their transcriptome; we removed these samples from the following analyses, as they represented outliers, possibly related to intrinsic molecular characteristics or even to RNA processing. Thus, only samples with a high expression of circRNAs (*n* = 1018) were studied further, using the median of total log_2_ circRNA RPM counts as a threshold.

### 2.5. Functional Characterization of the circRNAs Encoding Proteins

The genes harboring circRNAs dysregulated in cancer and potentially translated were analyzed using PantherDB (http://pantherdb.org/, accessed on 15 June 2022). Correction for multiple testing was performed using the false discovery rate (FDR), with a threshold level of 0.05.

### 2.6. Mass Spectrometry Identification of Novel Peptides Derived from Coding circRNAs

To identify peptides corresponding to the novel N- or C-terminus predicted from circORFs, we used PeptideAtlas (http://www.peptideatlas.org/, build Human 2021-01, accessed on 1 August 2019), a compendium of peptides collected by tandem mass spectrometry experiments from humans and other organisms [21,22]. FASTA36 (version 36.3.8 h, accessed on 1 August 2019) was used for identifying circORF peptides in the PeptideAtlas database. Additionally, BLASTP 2.13.0+, and RefSeq were used to re-evaluate any peptides identified from Peptide Atlas.

### 2.7. Prediction of Internal Ribosome Entry Sites (IRES) in circRNAs with Coding Potential

IRESpy (https://irespy.shinyapps.io/IRESpy/, accessed on 20 October 2022), a high-throughput IRES tool based on the XGBoost model, was used to predict IRES in circRNAs with a novel primary structure and/or novel domain composition. Table S3 shows the circRNAs with a predicted probability (prob_IRES) higher than 0.3.

### 2.8. Identification of m6A Sites in Coding-circRNA Sequences

To investigate the overlapping regions, we intersected the chromosomal coordinates of the circRNAs differentially expressed in the Pan-cancer dataset (Table S12) with N6-adenosine methylation (m6A) peak information sourced by REPIC RNA Epitranscriptome Collection (https://repicmod.uchicago.edu/repic/download.php, dataset name: all human m6A peak information, Assembly:hg38, accessed on 27 February 2023).

## 3. Results

### 3.1. Cancer circRNAs Potentially Encode Novel Proteins

**Figure 2:**
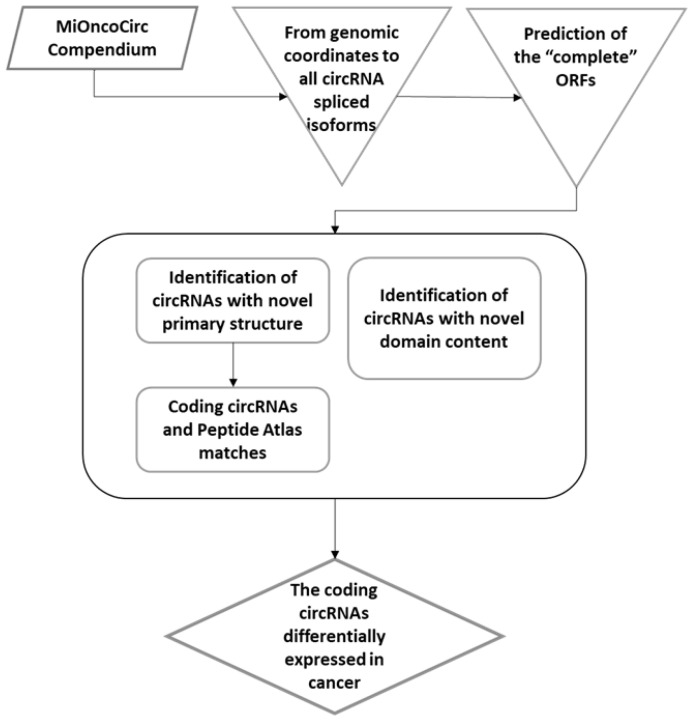
The flow chart representation of the study. The diagram synthesizes the bioinformatics analysis flow, which led to the identification of the circRNAs with novel coding potential (structurally divergent proteins from those encoded by the linear mRNAs) and differentially expressed in a set of human cancer types.

This study aimed to understand whether circRNAs expressed in cancer have a coding potential. We looked for the most relevant alterations compared with the canonical linear (cognate) isoforms. We hypothesized that circRNAs of interest in cancer should encode unique and novel peptide/protein, structurally and functionally different from the canonical proteins, encoded by the same loci. As function-related changes, we considered those including full or partially novel polypeptides (primary structure), as well as modifications of the canonical domain structure. The bioinformatics procedure, leading to the identification of such circRNAs, is illustrated in Figure 2.

We predicted the proteins encoded from circRNAs and considered those at least 50 aa residues long. Then, we looked for novel sequences among these circORFs-encoded proteins: for each circRNA, we focused on the longest circORF proteins (*n* = 4361) bearing a partial, but not complete, overlap (E-value lower than 1.0 × 10^−10^) with canonical cognate counterparts (from CCDS). Because of their conformation, the ORFs with partial overlap with the cognate CCDS sequences were bearing novel sequences either at the N-terminus, C-terminus, or at both. Most of these novel protein-coding circRNAs started at the canonical Methionine (canonicalMet), (*n* = 3363); a smaller number started at an internal Met (*n* = 824) (compared with the parental protein), while the remaining (*n* = 174) started at a novel 5′ Met, thus encoding for a novel N-terminus (N-term). As far as the C-terminus, most of the predicted circORFs encoded for novel sequences (*n* = 3865), while a few were terminated prematurely (prematureTerm) leading to a truncated protein (*n* = 223), or had the canonical stop codon (canonicalSTOP) (*n* = 273). Overall, the largest portion of circORFs had a canonical Met start and novel primary structure at the C-terminus (C-term). Only a small percentage possessed both novel N- and C-terminus (*n* = 115) (Figure S1).

The distribution of the length for the predicted novel peptide extensions, individually, at the N- and at C-terminus, alongside the descriptive statistics, are plotted in Figure S2. The average length of the novel peptides was slightly higher for N-terminus (mean = 24.1 aa) than the C-terminus (mean = 17 aa). 

In addition, we looked for specific differences in the domain structure of circRNA-encodable proteins. Most of them shared the exact domain structure with their linear isoforms. The most frequent structural alteration was the loss of domains compared with the cognate linear mRNAs; indeed, 931 circORF proteins lacked one or more domains, whereas 120 showed a different domain order, 50 included an extra domain, 58 presented concurrent domain loss and the inclusion of extra domains, and 20 circORF proteins had a completely novel domain structure (Figure S3 and Tables S4–S9).

In a different approach, other investigators considered IRES in the circRNA sequence as necessary for efficient circRNA translation [23,24]. Accordingly, we used IRESPy [25] to predict IRES probability in the circRNAs with a coding potential and/or a novel domain composition. Among the 4664 circRNAs considered, 59 had an IRES probability higher than 0.3 (Table S3). The translation of circRNAs is a cap-independent event so it can rely on IRES or N6-methyladenosine (m6A) RNA modifications. Besides considering the IRES elements, we checked if any of the coding circRNAs overlapped with the m6A sites. Over the 183 total circRNAs differentially expressed, we identified 156 of them as overlapping with known m6A sites. As described by Wen S.Y. et al., m6A sites were enriched in circRNAs and functioned as IRES-like elements, so just a single m6A modification could drive the translation of these coding circRNAs [26].

To experimentally validate our results, we looked for the presence of the novel circRNA-specific N- and C-terminus, separately considered, among the peptides from a human organism sequenced by tandem mass spectrometry from the PeptideAtlas database. Among the total of 82 peptides, 70 were novel carboxy peptides and 12 were novel amino peptides, perfectly overlapped with sequences reported in the PeptideAtlas database (Table S10). Regarding the tissue distribution of the circRNA-peptide matching in PeptideAtlas, we also reported their localization and the number of observations in Table S10. 

We highlighted five matched peptides also overlapping with the ORF of differentially expressed circRNAs encoded by SUCO, TBC1D31, PTBP3, GANAB, and RNF13 genes.

### 3.2. Expressed Coding circRNAs with Novel Coding Properties Are Involved in Cancer Pathways

**Figure 3:**
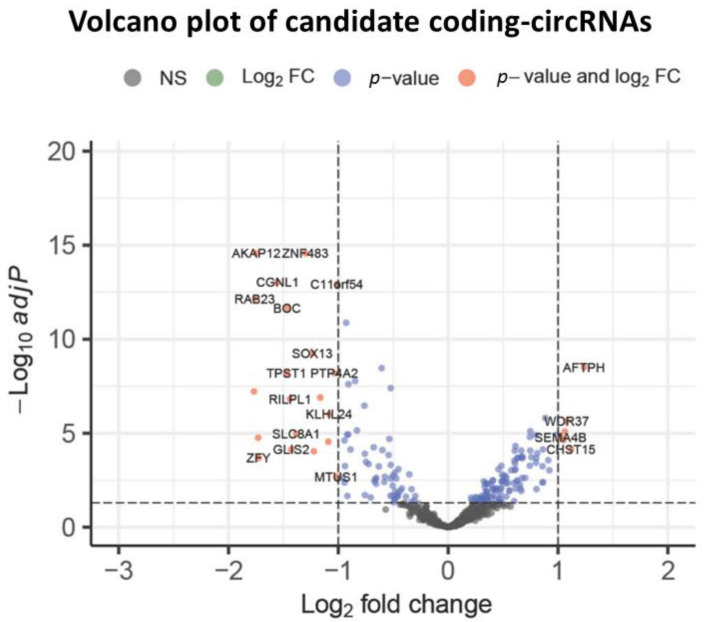
Summary of the RNA-seq results. The volcano plot shows the differential expression of the candidate circRNA proteins in the cancer samples versus the control data sets. Orange points mark the genes with significantly increased or decreased expression (adjusted *p* value < 0.05). The x-axis shows log_2_ fold changes in expression and the y-axis the log_10_ adjusted *p* value of a gene being differentially expressed. A number of genes was filtered out the list of candidate coding circRNAs because of the log_2_ fold changes < 1 as absolute value (blue points) and adjusted *p* value > 0.05 (black points).

In this further step, we specifically investigated the expression profiles of coding circRNAs, with either novel amino-/carboxy-terminus or domain structures, in 1018 human samples from the MiOncoCirc compendium. Finally, 629 coding circRNAs had a highly variable expression across the remaining samples (IQR > 0.5) (Table S11). To investigate their possible cancer roles, we performed differential expression analysis identifying 183 coding circRNAs deregulated in cancer versus the control tissues. The Benjamini–Hochberg adjustment was applied for multiple testing (Figure S4 and Table S12) and the deregulated coding circRNAs were visualized using a volcano plot (Figure 3).

Among these, AKAP12 and ZNF483 emerged as the most differentially down-regulated coding circRNAs whereas AFTPH, CHST15 and WDR37 were differentially up-regulated in the pan-cancer RNA-Seq dataset. 

Most of these coding circRNAs (*n* = 121) harbored N- and/or C-terminal novel sequence, 9 displayed only novel domain content, while 28 had both types of structural changes. Furthermore, 12 circRNAs started from an internal methionine (shorter N-terminal) and/or had premature termination, leading to truncated proteins without additional extra sequences. In Figure S5, we provide two alignment examples of the novel peptides at the C-term of FLI1 and HIPK3 coding circRNAs with the RefSeq protein database. Here, we highlight, in red, the novel sequence of circRNAs which overlap with the proteome of other organisms different from homo sapiens. We underline that the peptides overlapping with predicted new ORFs do not align anywhere else in the genome because they are specific. circRNAs potentially coding novel polypeptides could, in principle, bear novel functional roles. For example, a circRNA protein missing a domain could act as a dominant negative, or display altered cellular localization. Therefore, we studied the gene ontology (GO) and the molecular features of these 183 circORFs deregulated in cancer. We interrogated PantherDB [27] to perform an over-representation analysis of the circORF genes. The results showed a significant (BH corrected *p* value < 0.05) over-representation for biological processes, molecular functions, cellular components, and reactome pathways (Table 1 and Table S13).

**Figure 4:**
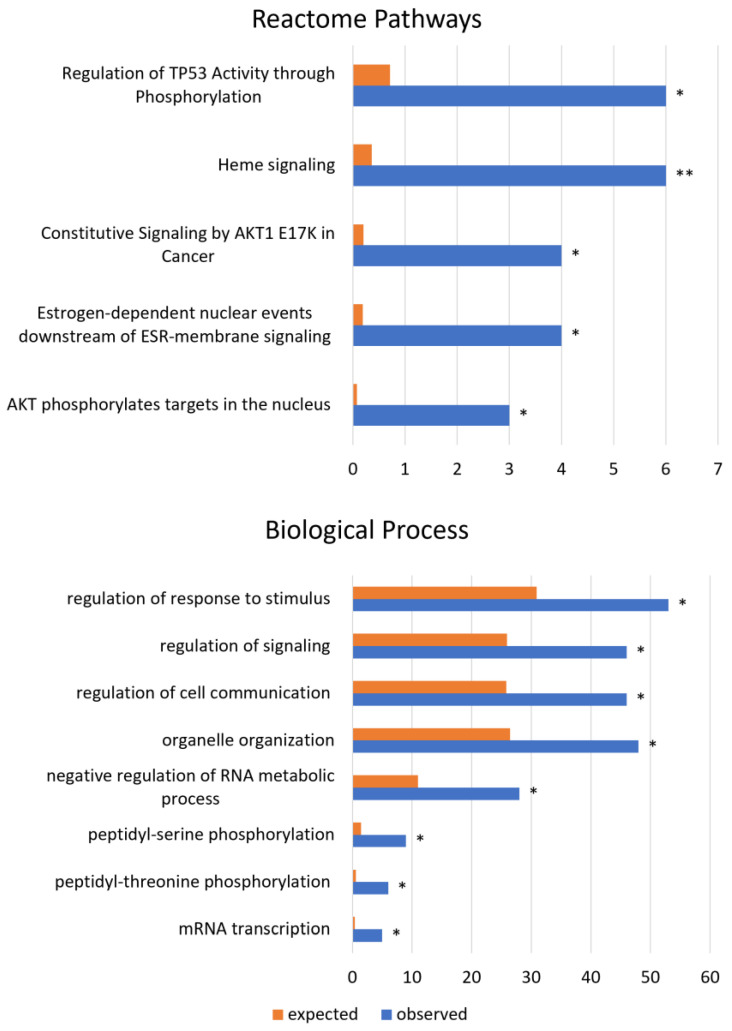
GO analysis: Reactome pathways and biological process for circRNAs novel peptides in cancer. The figure shows the statistical overrepresentation of the biological processes altered in cancer for the differentially expressed circRNAs with a novel coding potential (*n* = 183). The orange bars indicate the expected number of genes predicted for each category, while the blue bars show the observed number of genes in each of them. *p* values are adjusted using FDR correction: 0.05 < * < 0.01; 0.01 < ** < 0.001.

Among the reactome pathways associated with circORFs deregulated in cancer, we listed the regulation of TP53 activity through phosphorylation, heme signaling, and constitutive signaling by the AKT1 E17K mutation. The most significant biological processes overrepresented by coding circRNAs were the regulation of response to stimulus, signal transduction, cell communication, organelle organization, and peptidyl phosphorylation, which are illustrated in Figure 4.

Furthermore, we found molecular functions such as protein kinase activity; DNA binding; and cellular components, such as nuclear speck and rough endoplasmic reticulum, among those mapped by the circRNAs to have a coding potential and be differentially expressed in human cancer.

### 3.3. The Coding circRNAs Differentially Expressed in Cancer Are Also Involved in AML

In a previous study [6], we profiled the total RNA transcriptome of 345 patients affected by cytogenetically normal AML (CN-AML), identifying a set of circRNAs with prognostic value. We hypothesized that some AML prognostic circRNAs could also be among the cancer coding circRNAs we evidenced above. Interestingly, 24 circRNAs associated with prognosis in CN-AML (Table 2) were indeed present in the pan-cancer coding circRNAs profile, with 8 of them being differentially expressed in cancer (adjusted *p* value < 0.05). We performed a two tailed Fisher’s test to demonstrate that the presence of 8 coding circRNAs (indicated with an asterisk in Table 2) exceeded that expected by random association (*p* value < 0.001).

## 4. Discussion

Since their discovery, there has been much debate about the cellular roles of circRNAs. To date, an increasing number of reports have described the differential expression of circRNAs in normal and tumor samples [5], reporting their functions as “sponges” of microRNA [28,29] or decoys of protein [30,31]. A few studies have demonstrated that circRNAs act as messenger RNAs to be translated by ribosomes [32]. The aim of our study was to explore the latter possibility and to systematically investigate the coding potential of more than 160,000 species of circRNA expressed in cancer. For this purpose, we leveraged data obtained from the MiOncoCirc pan-cancer compendium, produced by total and exome capture RNA-seq. Using a bioinformatics approach, we predicted all of the polypeptides encodable from circORFs. Then, we focused on the predicted circORF proteins that, with respect to their same gene (or cognate) linear isoforms, had novelty in the (i) primary structure and/or (ii) domain structure. Critically, we highlighted those circRNAs with predicted polypeptides starting at the same Methionine as their cognate mRNAs (canonicalMet), and thus expected to be de facto translatable by the 5′-flanking site, where the protein translation machinery enables ribosomes binding. Overall, we identified 3723 circORFs potentially encoding for novel peptides at the C- or N-terminus in the absence of domain alterations, and 1179 such circRNAs encoding for proteins with a novel domain structure. Among the coding circRNAs considered, 59 of them also had an IRES probability higher than 0.3. To further pinpoint highly relevant coding circRNAs in cancer, we performed a differential expression analysis in 1018 human cancers, cell lines, and control samples and identified 183 genes that encode for circRNAs. In particular, ZNF483 and AKAP12 emerged as the strongest down-regulated circRNAs in cancer. Interestingly, the loss of AKAP12 and ZNF483 expression were described in prostate cancer [33] and acute lymphoblastic leukemia [34], respectively; on the other hand, AFTPH was the most up regulated circRNA in our analysis. This gene has been reported as a potential target and prognostic factor, because of its effects on proliferation, in different types of cancer such as breast cancer, diffuse large B-cell lymphoma, lung squamous cell carcinoma, and pancreatic adenocarcinoma, in which it is over-expressed [35]. Nevertheless, there are no studies to date about the role of these circRNAs. 

Conversely, the circRNAs encoded by CHST15, WDR37, and SOX13, and their role in cancer have been previously delineated in the literature. CircCHST15 displayed a high expression in our study, according to the findings of Gui C.P. and Yang J., in clear cell renal cell carcinoma [36] and lung cancer [37], respectively. This circRNA is associated with proliferation, migration, invasion, and immune escape, through the activation of PD-L1. Thus, the over-expression of circCHST15 is an indication of a poor prognosis. CircWDR37, instead, exerts its role activating the PKR cascade, which results in the promotion of NF-κB activation, proliferation, and senescence-driven metastasis. In fact, experimentally lowered circWDR37 is correlated with chemotherapy response and favorable survival in nasopharyngeal carcinoma patients treated with gemcitabine or cisplatin [38]. Indeed, it could represent a possible therapeutic target. Finally, circSOX13 was recently found to be up-regulated in lung cancer [39] and its knockdown showed an inhibition of proliferation, invasion, and migration, also revealing that it might reduce cisplatin resistance. In contrast, in our analysis, circSOX13 exhibited a log Fold Change of −1.24 and an average expression of −4.75 as seen in Pedraz-Valdunciel’s experiments, the circRNA was downregulated even at the early stages of non-small cell lung cancer [40] and, as a result, it has the potential to be a cancer biomarker. We also found that 156 coding circRNAs differentially expressed in cancer overlapped m6A sites, a strong indicator of their potential translation. We highlight that the coding circRNAs with a differential expression in cancer were associated with (i) biological processes such as regulation of signaling and protein phosphorylation, (ii) molecular functions such as DNA binding and protein serine kinase activities, (iii) cellular components such as nuclear specks, and (iv) Reactome Pathways such as “constitutive signaling by AKT1 E17K in cancer” and “heme signaling”. Consistent with our working hypothesis, we found that some of the coding circRNAs in our list had already been reported in the literature. Strikingly, the β-catenin-370 aa isoform [15] was independently identified in our screen as a circORF with a novel C-terminus (with six extra residues). A second previously reported circRNA protein, FBXW7-185 [12,41], was also present among our circORFs, with a novel C-terminus (Table S11). Another 24 circRNAs were associated with prognosis in acute myeloid leukemia [6], and were also included in the pan-cancer coding circRNAs profile. These multiple findings converged to support a strong functional role for the coding circRNAs in cancer.

Finally, we aimed to look for experimental validation of our bioinformatics investigation. We underline that it is potentially challenging to identify the peptides derived from circRNA translation, because there are not many within the proteins and they might not be readily detectable with the usual analytical techniques, for example using liquid chromatography–tandem mass spectrometry [42]. We interrogated the mass spectrometry PeptideAtlas database to identify any match with circORFs’ peptides. More than 80 peptides generated from mass spectrometry (Table S10) were compatible with circORF specific amino acid sequences, corroborating our initial hypothesis and our circORF findings. We would also like to evidence that very few peptides encoded by circRNAs, currently about 13, have been validated in the literature [26], such as AKT3 [43], FBXW7-185aa [12], PINT-87aa [13], SHPRH-146aa [14], circPPP1R12A [44], and, more recently, CircSEMA4B [45].

As circRNA could display its function not only through its possible translated peptide, but also through its interaction with other molecules (ribosomes or miRNAs), and acting as a non-coding RNA molecule [46,47], in the future, both ways of action using antibodies especially designed and synthesized to be selective for the circular encoded peptide [12], and specific antisense oligonucleotide or knockout systems to suppress the transcribed circRNA form must be investigated [48].

## 5. Conclusions

Our study aims to contribute to unveiling the presence of a sizable group of circRNAs that have the potential to generate novel protein components in the cellular circuitry, specifically in cancer. Whether these coding circRNAs might constitute a core of more stable mRNA forms, be mass regulated by somatic mutations in the splicing machinery genes, and really impact key cancer pathways remains to be experimentally determined.

## Figures and Tables

**Table 1:** Gene Ontology analysis for circRNAs novel peptides in cancer. The table illustrates the GO category significantly mapped and over-represented by the coding circRNAs differentially expressed in cancer. We used PantherDB to perform a statistical over-representation GO analysis. Fisher’s exact test was applied to evaluate the significance of the observed/expected gene ratio for each GO category and the BH method to adjust the *p* values (FDR < 0.05). MF, molecular function; BP, biological process; CC, cellular component; RP, reactome pathway; pol II, RNA polymerase II; obs, observed; exp, expected.

GO Categories	GO	Obs	Exp	Fold	*p* Value	FDR
AKT phosphorylates targets in the nucleus	RP	3	0.08	38.85	1.19 × 10^−4^	3.31 × 10^−2^
Estrogen-dependent nuclear events downstream of ESR-membrane signaling	RP	4	0.19	21.58	5.89 × 10^−5^	2.45 × 10^−2^
Constitutive Signaling by AKT1 E17K in Cancer	RP	4	0.2	19.92	7.79 × 10^−5^	2.77 × 10^−2^
Heme signaling	RP	6	0.36	16.53	3.14 × 10^−6^	2.61 × 10^−3^
Regulation of TP53 Activity through Phosphorylation	RP	6	0.71	8.45	1.08 × 10^−4^	3.38 × 10^−2^
aryl hydrocarbon receptor binding	MF	3	0.07	43.16	9.23 × 10^−5^	3.78 × 10^−2^
protein serine kinase activity	MF	14	2.8	4.99	1.30 × 10^−6^	2.13 × 10^−3^
protein serine/threonine kinase activity	MF	14	3.34	4.2	9.06 × 10^−6^	6.35 × 10^−3^
protein serine/threonine/tyrosine kinase activity	MF	14	3.46	4.05	1.34 × 10^−5^	7.33 × 10^−3^
DNA binding	MF	39	19.3	2.02	1.57 × 10^−5^	7.68 × 10^−3^
rough endoplasmic reticulum	CC	5	0.63	7.9	5.53 × 10^−4^	4.70 × 10^−2^
transcription regulator complex	CC	15	3.91	3.84	1.20 × 10^−5^	1.63 × 10^−3^
nuclear speck	CC	11	3.19	3.45	4.41 × 10^−4^	4.09 × 10^−2^
centrosome	CC	14	4.87	2.87	4.57 × 10^−4^	4.05 × 10^−2^
cytosol	CC	65	42.04	1.55	9.16 × 10^−5^	1.04 × 10^−2^
mRNA transcription	BP	5	0.38	13.21	5.80 × 10^−5^	3.49 × 10^−2^
peptidyl-threonine phosphorylation	BP	6	0.58	10.36	3.72 × 10^−5^	3.43 × 10^−2^
peptidyl-serine phosphorylation	BP	9	1.42	6.33	1.85 × 10^−5^	3.23 × 10^−2^
negative regulation of RNA metabolic process	BP	28	11.02	2.54	5.46 × 10^−6^	2.14 × 10^−2^
organelle organization	BP	48	26.44	1.82	2.44 × 10^−5^	3.19 × 10^−2^
regulation of cell communication	BP	46	25.81	1.78	5.71 × 10^−5^	3.58 × 10^−2^
regulation of signaling	BP	46	25.91	1.78	8.60 × 10^−5^	4.65 × 10^−2^
regulation of response to stimulus	BP	53	30.9	1.72	4.71 × 10^−5^	3.69 × 10^−2^

**Table 2:** Coding circRNAs associated with prognosis in CN-AML.

Gene	chr	Start	End	Annotation
ABHD2	chr15	89113724	89116521	C-term|canonicalMet
ANKRD12 *	chr18	9182381	9221999	C-term|canonicalMet|conservedStructure
ARAP2 *	chr4	36228581	36229645	C-term|canonicalMet|lackingDomain
CLNS1A	chr11	77619605	77625818	canonicalSTOP|conservedStructure|internalMet
CPSF6	chr12	69251128	69262562	canonicalSTOP|conservedStructure|internalMet
CSNK1G3	chr5	123545416	123557564	C-term|canonicalMet
FBXW7	chr4	152411302	152412529	C-term|canonicalMet
HIPK3 *	chr11	33286412	33287511	C-term|canonicalMet|conservedStructure
KLHL8	chr4	87195323	87195690	C-term|canonicalMet
MGA	chr15	41668827	41669958	C-term|canonicalMet|lackingDomain
NCOA2 *	chr8	70213902	70216764	C-term|canonicalMet|novelDomainStructure
OMA1	chr1	58506059	58539310	C-term|canonicalMet|conservedStructure
PCMTD1	chr8	51860844	51861246	C-term|canonicalMet|conservedStructure
PDE3B	chr11	14771936	14789242	C-term|internalMet
RELL1	chr4	37631384	37638504	canonicalSTOP|internalMet
RNF13 *	chr3	149846010	149921227	C-term|canonicalMet|conservedStructure
RNF220	chr1	44411980	44412722	C-term|canonicalMet
RSRC1	chr3	158122102	158123991	C-term|canonicalMet
SATB1 *	chr3	18378169	18420991	C-term|canonicalMet|lackingDomain
SHOC2	chr10	110964124	110985765	lackingDomain
SLC38A1	chr12	46229152	46243314	C-term|canonicalMet|conservedStructure
SLC8A1 *	chr2	40428472	40430304	C-term|canonicalMet|conservedStructure
XPO1 *	chr2	61522610	61533903	C-term|canonicalMet|conservedStructure
ZBTB44	chr11	130260855	130261929	C-term|canonicalMet|lackingDomain

* differentially and significantly expressed in pan-cancer dataset than normal samples; chr: chromosome (*p* value < 0.001).

## Data Availability

Not applicable.

## Supplementary Materials

The following supporting information can be downloaded at: https://www.mdpi.com/article/10.3390/biology12040493/s1. Figure S1: The percentage of circORFs characterized by novel or unexpected terminuses. The pie chart illustrates the number and the relative percentage of the circORFs with novel or unexpected combinations of terminuses compared to all linear counterparts. The circORFs annotated as “canonicalMet|canonicalSTOP” are not included in the pie chart. Figure S2: Density of the amino- and carboxy-terminus lengths from circRNAs with coding potential and the relative statistics. Figure S3: Domain structure of predicted circRNA proteins compared with the parental linear isoforms. The pie chart illustrates the number and the percentage of circRNAs with novel domain composition. The grey section represents the circRNAs with identical structure to the linear counterparts. Figure S4: CircRNAs, predicted to code for novel proteins, display differential expression in cancer and normal samples. The heatmap analysis shows the expression profiles of 183 circRNAs in 1018 cancer, cell lines and normal human samples from the MiOncoCirc compendium. Figure S5: Alignment examples of the novel peptides at C-term of the coding-circRNA FLI1 and HIPK3 with the RefSeq protein database by protein BLAST tool. Table S1: Types of cancer representation in pan-cancer dataset “MiOncoCirc”. Table S2: Sample annotations. Table S3: Prediction of internal ribosome entry site in circRNAs with coding potential. Table S4: CircRNAs with an identical domain structure than linear isoforms. Table S5: CircRNA with lacking domain than linear isoforms. Table S6: CircRNAs with a different order of domains than linear isoforms. Table S7: CircRNAs with lacking and extra domains than linear isoforms. Table S8: CircRNAs with extra domains than linear isoforms. Table S9: CircRNAs with a novel domain structure than linear isoforms. Table S10: CircRNAs with peptides (at least 10 aa long) matched in PeptideAtlas. Table S11: CircRNAs with highly variable expression across cancer and control human samples (IQR > 0.5). Table S12: CircRNAs differentially expressed in pan-cancer dataset “MiOncoCirc”. Benjamini Hochberg adjusted *p* value < 0.05. logFC, log fold change; AveExpr, average expression. Table S13: GO analysis of genes which encode for circRNAs with coding potential and differentially expressed in a pan-cancer dataset.
