# Supplementary material for: Circular RNAs Could Encode Unique Proteins and Affect Cancer Pathways
Source: Biology (Basel). 2023 Mar 24;12(4):493. doi: 10.3390/biology12040493 (PMC10135897; doi:10.3390/biology12040493)
Supplement: Supplementary file 1 [file biology-12-00493-s001.zip › biology-2153287-Figure S2.pdf]

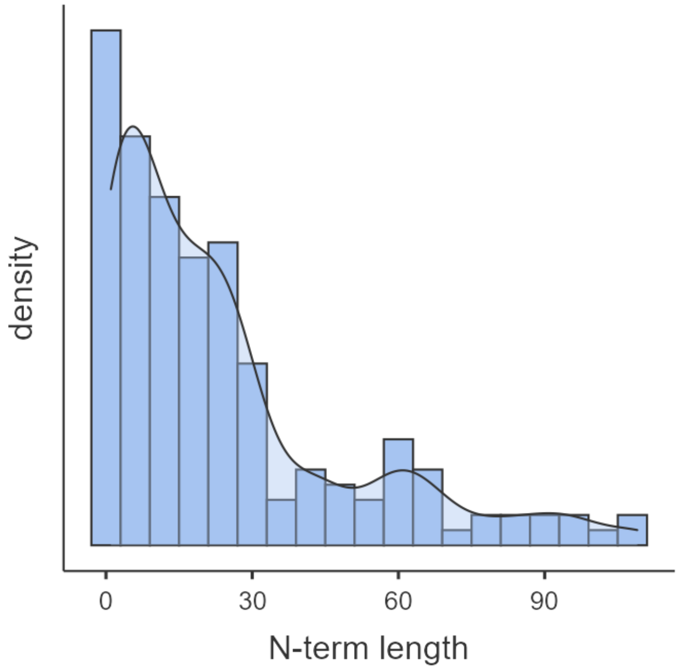

N-term length	
Valid	174
Mean	24.1
Median	16.0
Std. Deviation	25
Minimum	1
Maximum	109

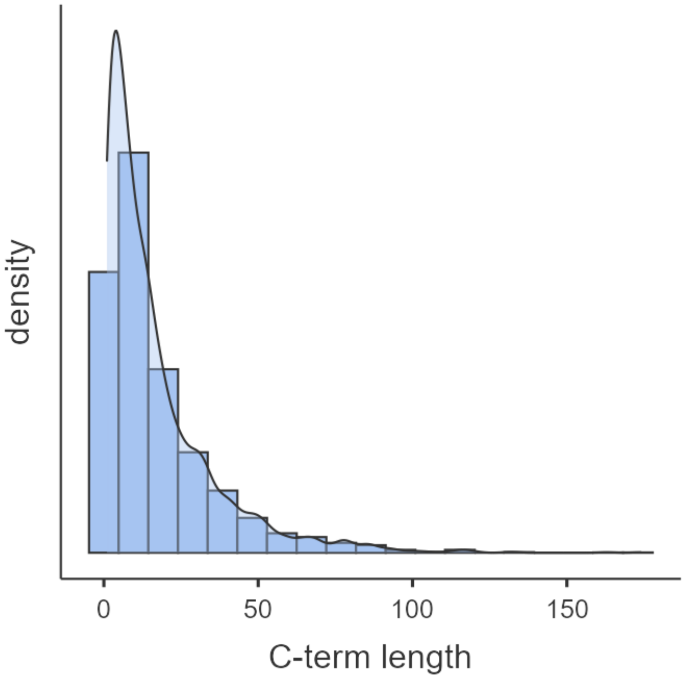

C-term length	
Valid	3865
Mean	17.0
Median	11.0
Std. Deviation	18.6
Minimum	1
Maximum	174
