# Supplementary material for: Circular RNAs Could Encode Unique Proteins and Affect Cancer Pathways
Source: Biology (Basel). 2023 Mar 24;12(4):493. doi: 10.3390/biology12040493 (PMC10135897; doi:10.3390/biology12040493)
Supplement: Supplementary file 1 [file biology-12-00493-s001.zip › biology-2153287-Figure S5.pdf]

Circ-protein FASTA: ENST00000281428.12_chr11_128758114_128782023_FLI1 | **Annotation:** C-term|canonicalMet|lackingDomain

MEGGLAGERARESPVDCSVSKCSKLVGGGESNPMNYNSYMDEKNGPPPPNMTTNERRVIVPADPTLWTQEHVRQWLEWAI KEYSLMEIDTSFFQNMDGKELCKMNKEDFLRATTLYNTEVLLSHLSYLRESSLLAYNTTSHTDQSSRLSVKEGGSVGGERRPVPL*

Parental protein FASTA: ENSP00000281428.8 per chromosome:GRCh38:11:128693916:128813166:1 gene:ENSG00000151702.17 transcript:ENST00000281428.12

MEGGLAGERARESPVDCSVSKCSKLVGGGESNPMNYNSYMDEKNGPPPPNMTTNERRVIVPADPTLWTQEHVRQWLEWAI KEYSLMEIDTSFFQNMDGKELCKMNKEDFLRATTLYNTEVLLSHLSYLRESSLLAYNTTSHTDQSSRLSVKEDPSYDSVRRGAWG NNMNSGLNKSPPLGGAQTISKNTEQRPQPDPYQILGPTSSRLANPGSGQIQLWQFLLELLSDSANASCITWEGTNGEFKMTDP DEVARRWGERKSKPNMNYDKLSRALRYYYDKNIMTKVHGKRYAYKFDFHGIAQALQPHPTESSMYKYPSDISYMPSYHAHQQ KVNFVPPHPSSMPVTSSSFFGAASQYWTSPTGGIYPNPNVPRHPNTHVPSHLGSYY

Pirin family protein [Streptomyces sp. CB03234]

Sequence ID: WP_073756648.1 Length: 330 Num. Of matches: 1

Score	Expect	Identities	Positives	Gaps
**32.9 bits(70)**	5.9	**10/10(100%)**	**10/10(83%)**	**0/10(0%)**
Query	2	GSVGGERRPVPL<br>GSVGGERRPVPL	11	
Sbjct	231	GSVGGERRPVPL	240	

Uncharacterized protein LOC107875993 [Capsicum annuum]

Sequence ID: XP_016578424.1 Length: 418 Num. Of matches: 1

Score	Expect	Identities	Positives	Gaps
**32.5 bits(69)**	8.3	**10/11(91%)**	**10/11(90%)**	**0/11(0%)**
Query	3	SVGGERRPVPL	13	
		SVGG RRPVPL		
Sbjct	259	SVGGARRPVPL	269	

Circ-protein FASTA: ENST00000303296.9_33286412_33328633_HIPK3.p1 **| Annotation**: C-term|canonicalMet

MASQVLVYPPYVYQTQSSAFCSVKKLKVEPSSCVFQERNYPRTYVNGRNFGNSHPPTKGSAFQTKIPFNRPRGHNFSLQTSAVV
LKNTAGATKVIAAQAQQAHVQAPQIGAWRNRLHFLEGPQRCGLKRKSEELDNHSSAMQIVDELSILPAMLQTNMGNPVTVV
TATTGSKQNCTTGEGDYQLVQHEVLCSMKNTYEVLDFLGRGTFGQVVKCWKRGTNEIVAIKILKNHPSYARQGQIEVSILARLST
ENADEYNFVRAYECFQHRNHTCLVFEMLEQNLYDFLKQNKFSPLPLKVIRPILQQVATALKKLKSLGLIHADLKPENIMLVDPVR
QPYRVKVIDFGSASHVSKTVCSTYLQSRYYRAPEIILGLPFCEAIDMWSLGCVIAELFLGWPLYPGALEYDQVWPHKSWSTHHM
FIKLSQVPFVV*

Parental protein FASTA: ENSP00000304226.4 pep chromosome:GRCh38:11:33257381:33357023:1 gene:ENSG00000110422.12 transcript:ENST00000303296.9

MASQVLVYPPYVYQTQSSAFCSVKKLKVEPSSCVFQERNYPRTYVNGRNFGNSHPPTKGSAFQTKIPFNRPRGHNFSLQTSAVV
LKNTAGATKVIAAQAQQAHVQAPQIGAWRNRLHFLEGPQRCGLKRKSEELDNHSSAMQIVDELSILPAMLQTNMGNPVTVV
TATTGSKQNCTTGEGDYQLVQHEVLCSMKNTYEVLDFLGRGTFGQVVKCWKRGTNEIVAIKILKNHPSYARQGQIEVSILARLST
ENADEYNFVRAYECFQHRNHTCLVFEMLEQNLYDFLKQNKFSPLPLKVIRPILQQVATALKKLKSLGLIHADLKPENIMLVDPVR
QPYRVKVIDFGSASHVSKTVCSTYLQSRYYRAPEIILGLPFCEAIDMWSLGCVIAELFLGWPLYPGALEYDQIRYISQTQGLPGEQL
LNVGTKSTRFFCKETDMSHSGWRLKTLEEHEAETGMKSKEARKYIFNSLDDVAHVNTVMDLEGSDLLAEKADRREFVSLLKKML
LIDADLRITPAETLNHPFVNMKHLLDFPHSNHVKSCFHIMDICKSHLNSCDT.....PTKLSQYPYM

Uncharacterized protein LOC109682218 [Castor canadensis]

Sequence ID: XP_020012930.1 Length: 575 Num. Of matches: 1

Score	Expect	Identities	Positives	Gaps
**82.9 bits(188)**	8e-17	**23/23(100%)**	**23/23(100%)**	**0/23(0%)**
Query	1	VWPHKSWSTHHMFIKLSQVPFVV	23	
		VWPHKSWSTHHMFIKLSQVPFVV		
Sbjct	553	VWPHKSWSTHHMFIKLSQVPFVV	575	

PREDICTED: vescicle transport protein GOT1B [Raphanus sativus]

Sequence ID: XP_018481501.1 Length: 135 Num. Of matches: 1

Score	Expect	Identities	Positives	Gaps
**32.9 bits(70)**	28	**11/16(69%)**	**12/16(75%)**	**2/16(12%)**
Query	7	WSTHHMFIKLSQVPFV	22	
		WST MF LSQ+PFV		
Sbjct	103	WSTVKMF---LSQIPFV	116	
