# Supplementary material for: Circular RNAs Could Encode Unique Proteins and Affect Cancer Pathways
Source: Biology (Basel). 2023 Mar 24;12(4):493. doi: 10.3390/biology12040493 (PMC10135897; doi:10.3390/biology12040493)
Supplement: Supplementary file 1 [file biology-2153287-Figure S4.pdf › biology-2153287-Figure S4.pdf]

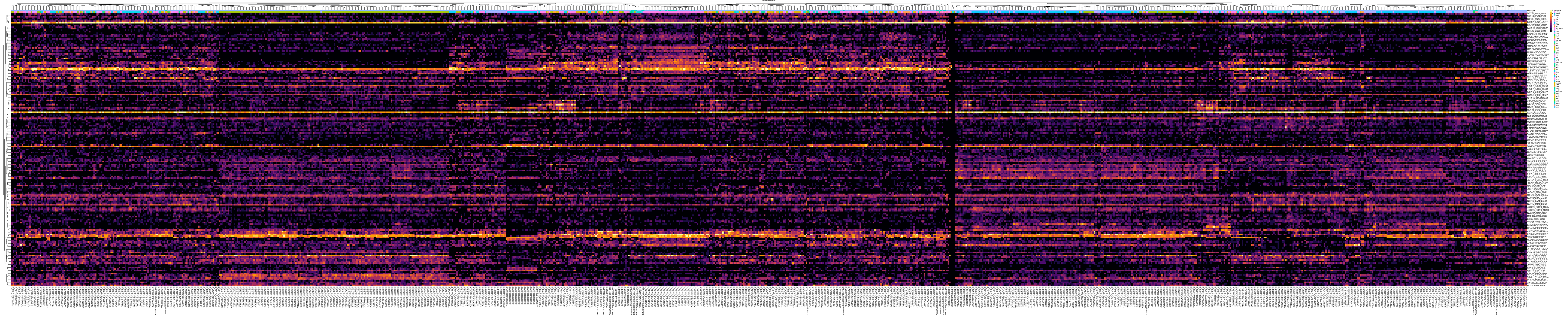
Correlation Heatmap
